# Frailty assessment tools predict perioperative outcome in elderly patients with endometrial cancer better than age or BMI alone: a retrospective observational cohort study

**DOI:** 10.1007/s00432-022-04038-6

**Published:** 2022-05-17

**Authors:** Katharina Anic, Friedrich Flohr, Mona Wanda Schmidt, Slavomir Krajnak, Roxana Schwab, Marcus Schmidt, Christiane Westphalen, Clemens Eichelsbacher, Christian Ruckes, Walburgis Brenner, Annette Hasenburg, Marco Johannes Battista

**Affiliations:** 1 Department of Gynecology and Obstetrics, University Medical Center of the Johannes Gutenberg University Mainz, Langenbeckstr. 1, 55131 Mainz, Germany; 2 Department of Geriatric Medicine, University Medical Center of the Johannes Gutenberg University Mainz, Langenbeckstr. 1, 55131 Mainz, Germany; 3 Department of Anesthesiology, University Medical Center of the Johannes Gutenberg University Mainz, Langenbeckstr. 1, 55131 Mainz, Germany; 4 Interdisciplinary Center Clinical Trials, University Medical Center Mainz, Langenbeckstr. 1, 55131 Mainz, Germany; 5 Management of the Scientific Laboratories, University Medical Center of Johannes Gutenberg University Mainz, Langenbeckstr. 1, 55131 Mainz, Germany

**Keywords:** Frailty, Perioperative, Endometrial cancer, Management, Outcome

## Abstract

**Objective:**

Five commonly used global health assessment tools have been evaluated to identify and assess the preoperative frailty status and its relationship with perioperative in-hospital complications and transfusion rates in older women with endometrial cancer (EC).

**Methods:**

Preoperative frailty status was examined by the G8 questionnaire, the Eastern Cooperative Oncology Group performance status, the Charlson Comorbidity Index and the American Society of Anesthesiologists Physical Status System, as well as the Lee-Schonberg prognostic index. The main outcome measures were perioperative laboratory values, intraoperative surgical parameters and immediately postoperative complications.

**Results:**

153 consecutive women ≥ 60 years with all stages of EC, who received primary elective surgery at the University Medical Center Mainz between 2008 and 2019 were classified with selected global health assessment tools according to their preoperative performance status. In contrast to conventional prognostic parameters like older age and higher BMI, increasing frailty was significantly associated with preoperative anemia and perioperative transfusions (*p* < 0.05). Moreover, in patients preoperatively classified as frail significantly more postoperative complications (G8 Score: frail: 20.7% vs. non-frail: 6.7%, *p* = 0.028; ECOG: frail: 40.9% vs. non-frail: 2.8%, *p* = 0.002; and CCI: frail: 25.0% vs. non-frail: 7.4%, *p* = 0.003) and an increased length of hospitalization were recorded. According to propensity score matching, the risk for developing postoperative complications for frail patients was approximately two-fold higher, depending on which global health assessment tool was used.

**Conclusions:**

Preoperatively assessed frailty significantly predicts post-surgical morbidity rates in contrast to conventionally used single prognostic parameters such as age or BMI. A standardized preoperative assessment of frailty in the routine work-up might be beneficial in older cancer patients before major surgery to include these patients in a prehabilitation program with nutrition counseling and physiotherapy to adequately assess the perioperative risk.

## Introduction

The complex concept of frailty and its increasing relevance in the ageing population require the recognition of the importance of global health assessment tools in everyday clinical practice (Lin et al. 2016; Birkelbach et al. 2019). Frailty, defined as a multidimensional clinical syndrome of age-associated decreased homeostatic reserves and function due to multiple organ systems, combines the dysregulation across various physiologic and molecular pathways with marked vulnerability to adverse health outcomes (Fried 2001; Rodríguez-Mañas et al. 2013). The “phenotype of frailty “results in diminished endurance and strength, a higher risk of falls, disability, hospitalization and mortality (Fried 2001). However, even this description from Fried et al. is the most cited, there is no commonly accepted uniform definition. Although there is no gold standard for detecting frailty, different global health assessment tools have been developed focusing on various aspects of health, especially in the preoperative setting (Table 1).

**Table 1:** Fragility assessment

Global health assessment tool	Description	Frailty-definition
***G8 questionnaire*** (G8 Score)	7 questions with different gradations in the predefined answers to various categories and the biological-calendaric age > Nutritional Status > Functional Status > Cognitive Status > Comorbidities	G8-frail: ≤ 14 points
***Eastern Cooperative Oncology Group*** (ECOG) ***performance status***	**0**: fully active, no performance restrictions **1**: strenuous physical activity restricted **2**: capable of only all self-care but unable to carry out any work activities **3**: capable of only limited self-care **4**: completely disables; cannot carry out any self-care **5**: death	ECOG-frail ≥ 2
***Charlson Comorbidity Index* (CCI)**	16 conditions with different weighting from the following categories > Cardiac diseases > Vascular diseases > Pulmonary diseases > Liver diseases > Metabolic disorders > Cancer in history	CCI-frail = 3
***American Society of Anesthesiologists Physical Status System*** (ASA PS)	Class 1: No systemic disturbance Class 2: mild systemic disturbance Class 3: serve systemic disturbance Class 4: extreme systemic disturbance Class 5: moribund patient who need emergency surgery, otherwise be graded as class 1/2 Class 6: moribund patient who need emergency surgery, otherwise be graded as class 3/4	ASA PS-frail ≥ 3
***Lee Schonberg prognostic index*** (Lee-Index)	Life span calculator, which measures the 4-year mortality using a combination of 15 questions from key health outcome categories: > Cardiac diseases > Pulmonary diseases > Vascular diseases > Cancer diseases > Daily life performance evaluation	Lee-frail: 4-year mortality ≥ 20% (Lee-Index ≥ 8 points)

Bold written words: used global health assessment tools and their individual classification system

As the current world population is getting older, there is an increased demand for special diagnostic and treatment algorithms specific to elderly cancer patients to offer multimodal oncological therapy regimens (Mohile et al. 2018). While increased age is often associated with more aggressive and advanced diseases (Bourgin et al. 2017; Emons et al. 2018), its independent role on mortality and morbidity remains controversial (Quaglia et al. 2009; Deiner and Silverstein 2011; El-Haddawi et al. 2002). The population older than 65 years is less likely to have all types of standardized oncological investigations resulting in an overall worse outcome in elderly cancer patients (Rauh-Hain et al. 2015). However, chronological age alone, as the single prognostic factor probably does not reflect the heterogeneity of the ageing process but because of the underrepresentation of elderly participants in clinical trials, only little evidence exists (Lewis et al. 2003).

Endometrial cancer (EC) is the most common gynecologic malignancy in developed countries with an incidence of one to two percent of females in the United States and 54,870 cases per year with 10,170 identified annual deaths (Bourgin et al. 2017; Schmidmayr and Dorn 2021; Chen and Berek 2021). EC primarily affects elderly women as age-standardized incidence rates peak after menopause, between the ages of 60 and 70 years, with a median age of 68 years at diagnosis (Sung et al. 2020). The main risk factors for EC include obesity, mostly in combination with other cardiovascular or metabolic diseases such as hypertension and diabetes mellitus, as well as chronic exogenous unopposed estrogen replacement treatments without progestin (Emons et al. 2018; Sorosky 2012). The keystone of treatment for EC, similar to other cancer types, is primary surgery for curative intended oncologic treatments and is considered a major stressor for patients. The extend of the surgical treatment depends on results of lymph node staging, the disease stage at diagnosis, various histological parameters, the health status of the patient and the present national guidelines (Morice et al. 2016). In general, frail patients are less likely to tolerate and adapt to radical, possibly multi-visceral surgical resections (Revenig et al. 2014; Li et al. 2018). Besides for postoperative complications, frailty also appears to be a risk factor for unplanned readmissions (Rothenberg et al. 2019; Robinson et al. 2011). As the incidence of frailty increases with age (25.5–56.1% in elderly patients vs. 6.9% in younger counterparts) (Amrock and Deiner 2014), frailty assessments become important especially in elderly cohorts with a higher incidence of cancer and an approximately 70% mortality (Sung, et al. 2020).

Therefore, the primary objective of this retrospective, observational cohort study was to evaluate the influence of frailty on perioperative complications in elderly endometrial cancer patients undergoing primary surgical treatment. Consequently, we assessed the predictive abilities of the preoperative frailty status evaluated by five broadly recognized global health assessment tools for the perioperative laboratory values and transfusion rates as well as postoperative complications.

## Methods

### Study population

This retrospective cohort analysis reports data from women older than 60 years of age treated consecutively at the University Medical Center Mainz—Johannes-Gutenberg University Mainz, Germany, between January 2008 and December 2019. All stages of EC were included. Standardized primary staging operations included hysterectomy with bilateral salpingo-oophorectomy, with or without pelvic and para-aortic lymph node resection, depending on tumor stage and the current national guidelines. The patients’ preoperative frailty status was retrospectively assessed based on the routine pre-surgical patient evaluation.

### Frailty assessments

In this study, the preoperative frailty status was assessed by five global health assessment tools (Table 1).

The *G8 questionnaire* (G8 Score) established in 2011 by Bellera et al. is a geriatric screening tool recommended by the International Society of Geriatric Oncology (SIOG) (Bellera et al. 2012). As a simple, time saving and reproducible questionnaire, the G8 Score aims to identify frail patients, who could benefit from a full Comprehensive geriatric assessment (CGA) after a two-step evaluation (Martinez-Tapia et al. 2017). The scoring system ranges from zero points (heavily impaired—G8-frail) to seventeen points (not impaired at all—G8-non-frail) with an established cut-off value of ≤ 14 points as an indicator of frailty (Hamaker et al. 2012).

The *Eastern Cooperative Oncology Group* (ECOG) *performance status* is one of the most commonly used methods to measure physiological reserves and functional status in cancer patients. The degree of functional impairment is divided into six categories, as a simplification of the in 1948 first described Karnofsky status. Five points, the maximum of the score, represent the clinical death, while a value of zero points represents normal unrestricted everyday activities prior to the disease (Rodin and Mohile 2007).

The *Charlson Comorbidity Index* (CCI) is an assessment of medical comorbidities to measure the estimated 1-year mortality and burden of disease, developed in 1987 (Charlson et al. 1987). Especially as a predictor of surgical mortality the sum of 16 different conditions according to ICD-10 codes has been validated (Molto and Dougados 2014).

The *American Society of Anesthesiologists Physical Status System* (ASA PS) is an international commonly used subjective rating system to categorize the preoperative overall medical health status of adult patients, first described in 1941 (Saklad 1941). The current ASA PS classification was proposed in 1961 by Dripps et al. (Fitz-Henry 2011), and was actually updated by Böhmer and colleagues (Böhmer et al. 2021). According to the inclusion criteria of the current study, emergency surgeries were excluded, thus ASA Class 5 and 6 were not included in the evaluation.

The *Lee-Schonberg prognostic index* (Lee-Index) has been “developed in 11,701 community-dwelling adults from the eastern, western and central United States who were interviewed in the Health Retirement Survey in 1998” (Lee et al. 2006). Depending on key health outcomes, the life expectancy calculator estimates the individual 4-year mortality through 15 selected questions.

### Data collection

General patient information was gathered from our hospital database, including biological tumor characteristics (tumor stage, histological grading and subtype), relevant lifestyle parameters like age, weight, smoking status and polypharmacy, as well as blood works and postoperative complications. Perioperative clinical and surgical complications were extracted according to the International Statistical Classification of Diseases and Related Health Problems (ICD-10) (WH Organization 2004) corresponding to the Veteran Affairs’ National Surgical Quality Improvement Program (NSQIP) (Khuri et al. 1998; Robinson et al. 2013). Long-term follow-up data were collected through telephone calls, written inquiries to the patients or their physicians, and by checking the available patient clinical records up to February 2021.

The G8 Score was determined retrospectively by adding the biological-calendar age to the Mini Nutritional Assessment (MNA) recorded by oncological nurses, trained in frailty assessment in cooperation with the geriatric department (Vellas et al. 1999; Rubenstein et al. 2001; Anic et al. 2021). The 4-year mortality rate routinely utilized and reproducibly estimated with the Lee-Index was modified afterwards by following the calculation without the cancer diagnosis. Relevant comorbidities were detected by the CCI, with the sum of sixteen different conditions according to ICD-10 codes (WH Organization 2004). ECOG was assigned individually by the operating surgeon during the consultation. The ASA PS was collected from all patients undergoing elective surgery at the anesthesia preoperative clinic of the Department of Anesthesiology.

### Statistical analyses

The manuscript was written in accordance with the STROBE-cohort checklist of the EQUATOR network reporting guidelines (Elm et al. 2007). Descriptive statistical analyses were performed with SPSS statistical software program, version 23.0 V5 R (SPSS Inc, Chicago, IL, U.S.A.) and StatalC 16 V5. All data analyses were carried out in an explorative approach.

Categorical data are given in absolute and relative frequencies. Continuous parameters are reported as mean ± standard deviation [SD] for normally distributed data and as medians and interquartile ranges (IQR) for non-parametric data. Differences in binary and ordinal variables between two independent groups were analyzed by the chi-square tests. To assess the usability in a clinical context, we defined the frailty status for each global health assessment tool by dichotomizing the values into two groups: frail and non-frail, respectively (Table 1). To preserve comparability, we furthermore dichotomized the study cohort into two groups, younger and older than the mean biological-calendaric age, as well as two groups with lower and higher BMI than the median, respectively. To investigate its impact on perioperative complications we used the exact chi-square test. A two-tailed *p* value < 0.05 was considered statistically significant. All analyses should be understood as explorative analyses, thus, no adjustment for multiple testing has been done.

To remove the simultaneous impact of frailty and other predictive factors as baseline confounder variables such as age and BMI especially for EC on the occurrence of perioperative events, we determined a propensity score model.

## Results

**Fig. 1:**
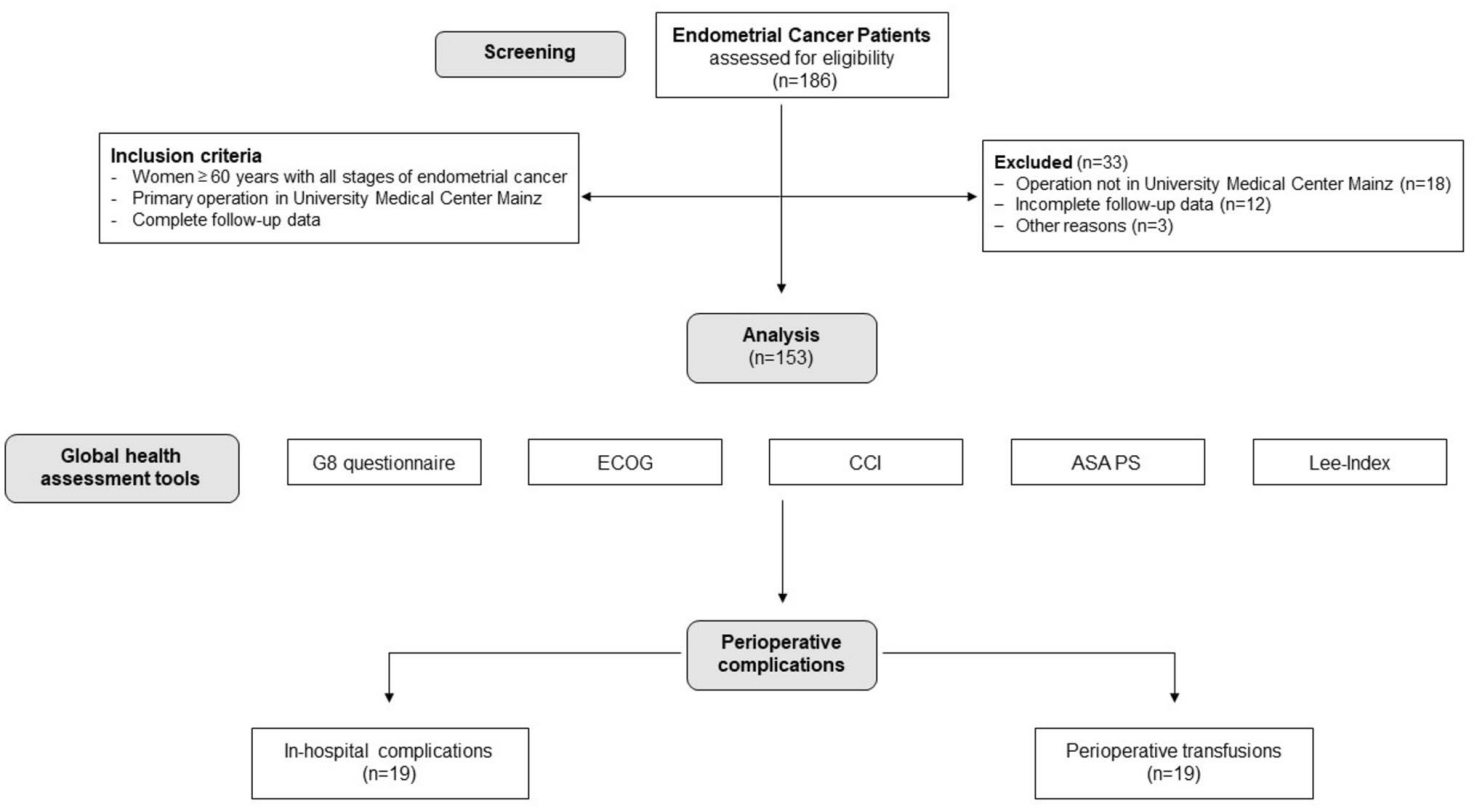
Consort statement

A total of 153 women aged older than 60 years (median 71 ± 7.4 years) were included in this study (for details see Fig. 1 and Table 2). Patients’ characteristics including tumor features, frailty status and lifestyle parameters, as well as postoperative events are presented in Table 2.

**Table 2:** Patients’ characteristics endometrial cancer (EC)

Parameter	(*n* = 153) *n* (%) (± SD)
**Tumor stage** (TNM, FIGO 2010)	
I Ia Ib	123 (80.9) 70 (46.1) 53 (34.9)
II	7 (4.6)
III IIIa IIIb IIIc1 IIIc2	12 (7.9) 2 (1.3) 3 (2.0) 5 (3.3) 2 (1.3)
IV IVa IVb	10 (6.6) 3 (2.0) 7 (4.6)
**Histological grading**	
G1	75 (49.7)
G2	46 (30.5)
G3	30 (19.9)
**Histological subtype**	
Adenocarcinoma	130 (85.0)
Others (endometrioid, squamous, mucinous)	23 (15.0)
**Surgical approach**	
Laparoscopic	49 (32.0)
Vaginal	29 (18.3)
Laparotomy	72 (49.7)
**Global health assessment tools**	
G8 questionnaire (G8 Score)	
G8 non-frail G8-frail	91 (61.1) 58 (38.9)
Eastern Cooperative Oncology Group (ECOG) performance status	
ECOG 0 ECOG 1 ECOG 2 ECOG 3	36 (38.7) 35 (37.6) 16 (17.2) 6 (6.5)
Charlson Comorbidity Index (CCI)	
CCI 1 CCI 2 CCI 3	37 (24.2) 72 (47.1) 44 (28.2)
American Society of Anaesthesiologists Physical Status System (ASA PS)	
ASA PS 1 ASA PS 2 ASA PS 3 ASA PS 4	4 (2.6) 59 (38.8) 87 (57.2) 2 (1.3)
Lee-Schonberg prognostic index (Lee-Index) [4-year mortality (%)]	
0 (< 10) 1 (10– < 20) 2 (20– < 30) 3 (> 30)	57 (39.0) 28 (19.2) 50 (34.2) 3 (7.5)
**Life style parameter**	
Mean age [years] (± SD)	71.00 (± 7.4)
Mean BMI [kg/m^2^]	30.19 (± 7.7)
Smoking-status	
Never smoker Active or former smoker	126 (90.6) 13 (9.4)
Polypharmacy (> 3 drugs)	76 (51.0)
**Perioperative events**	
Transfusion rate	19 (12.5)
In-hospital complications Pulmonary Thromboembolic Wound infection Multiple organ failure	19 (12.5) 5 (3.3) 3 (2.0) 8 (5.3) 3 (2.0)
Operative revisions	4 (2.6)

*EC* Endometrial Cancer, *n* number of patients, *SD* Standard Deviation, *TNM—FIGO* Tumor staging system—International Federation of Gynecology and Obstetrics, *G* Histological Grading*, ECOG* ECOG performance status, *ASA PS* ASA Physical Status Classification System, *G8 Score* G8 questionnaire, *G8 non-frail* G8 questionnaire > 14 points, *G8 frail* G8 questionnaire ≤ 14 points, *Lee-Index* Lee-Schonberg prognostic index, *BMI* Body Mass Index

Bold written words: analyzed main categories

Overall, data from a surgical point of view, that no differences were observed in intraoperative parameters between the frail and non-frail cohort, regardless of the used global health assessment tool, were published in a previous study (Anic et al. 2022). Neither the mean incision-suture with 142 ± 82.2 min nor the intraabdominal drainages rate of 64.7% or rate of operative revisions (2.6%) correlated with frailty status. One or more postoperative clinical in-hospital complications were observed in 19 patients (12.5%). Patients classified as frail determined by G8 Score, ECOG and CCI (Table 1), had significantly higher rates of preoperative anemia (hemoglobin < 10 g/dl) compared with non-frail patients, in contrast to the evaluation with ASA PS or Lee-Index (Table 3). Furthermore, the patients classified as frail received significantly more transfusions. Remarkably, age or BMI alone were not associated with anemia or the transfusion rates.

**Table 3:** Surgical characteristics in correlation with frailty status

*n* (%)	Preoperative hemoglobin [g/dl] < 10 10–12 > 12	Perioperative transfusions	Postoperative anaesthesiological intensive care treatment	Operative revisions	Clinical postoperative complications	Length of hospital stay ≥ 9 days	Death within 60 days
G8 questionnaire G8-frail	6 (10.5) 13 (22.8) 38 (66.7)	15 (25.9)	3 (5.2)	1 (1.7)	12 (20.7)	25 (43.1)	2 (10.5)
G8 non-frail	2 (2.2) 13 (14.4) 75 (53.3)	3 (3.3)	0 (0.0)	2 (2.2)	6 (6.7)	28 (30.8)	3 (23.1)
***p* value G8 Score**	**0.029**	** < 0.001**	**0.028**	**0.841**	**0.011**	**0.125**	**0.337**
Eastern Cooperative Oncology Group performance status ECOG-frail	3 (14.3) 7 (33.3) 11 (52.4)	7 (31.8)	3 (13.6)	1 (4.5)	9 (40.9)	13 (59.1)	5 (100.0)
ECOG non-frail	3 (4.3) 10 (14.3) 57 (81.4)	6 (8.5)	0 (0.0)	2 (2.8)	2 (2.8)	18 (25.4)	0 (0.0)
***p* value ECOG**	**0.025**	**0.006**	**0.002**	**0.688**	** < 0.001**	**0.003**	**0.026**
Charlson Comorbidity index CCI-frail	5 (11.4) 15 (34.1) 24 (54.5)	12 (27.3)	1 (2.3)	1 (2.3)	11 (25.0)	22 (50.0)	3 (6.8)
CCI non-frail	3 (2.8) 12 (11.2) 92 (79.3)	7 (6.5)	2 (1.8)	3 (2.8)	8 (7.4)	34 (31.2)	2 (1.8)
***p* value CCI**	** < 0.001**	** < 0.001**	**0.860**	**0.866**	**0.003**	**0.029**	**0.117**
Age < 71 ys	5 (6.0) 11 (13.3) 67 (80.7)	8 (9.6)	1 (1.2)	3 (3.6)	10 (11.9)	39 (40.2)	3 (20.0)
Age ≥ 71 ys	3 (4.4) 16 (23.5) 49 (72.1)	11 (57.9)	2 (2.9)	1 (1.4)	9 (13.2)	30 (53.6)	2 (11.8)
***p* value age**	**0.252**	**0.242**	**0.448**	**0.413**	**0.805**	**0.110**	**0.522**
BMI < 30 kg/m^2^	2 (2.4) 14 (16.5) 69 (81.2)	8 (9.3)	2 (2.3)	1 (1.2)	10 (11.8)	30 (34.9)	3 (13.0)
BMI ≥ 30 kg/m^2^	6 (9.2) 13 (20.0) 46 (70.8)	10 (15.4)	1 (1.5)	3 (4.5)	9 (13.6)	26 (39.4)	2 (22.2)
***p* value BMI**	**0.133**	**0.253**	**0.722**	**0.197**	**0.731**	**0.568**	**0.520**

*n* number of patients; bold written words: analyzed main *p* value categories, underlined words: analyzed main categories, bold written numbers: significant results (*p* < 0.05)

**Fig. 2:**
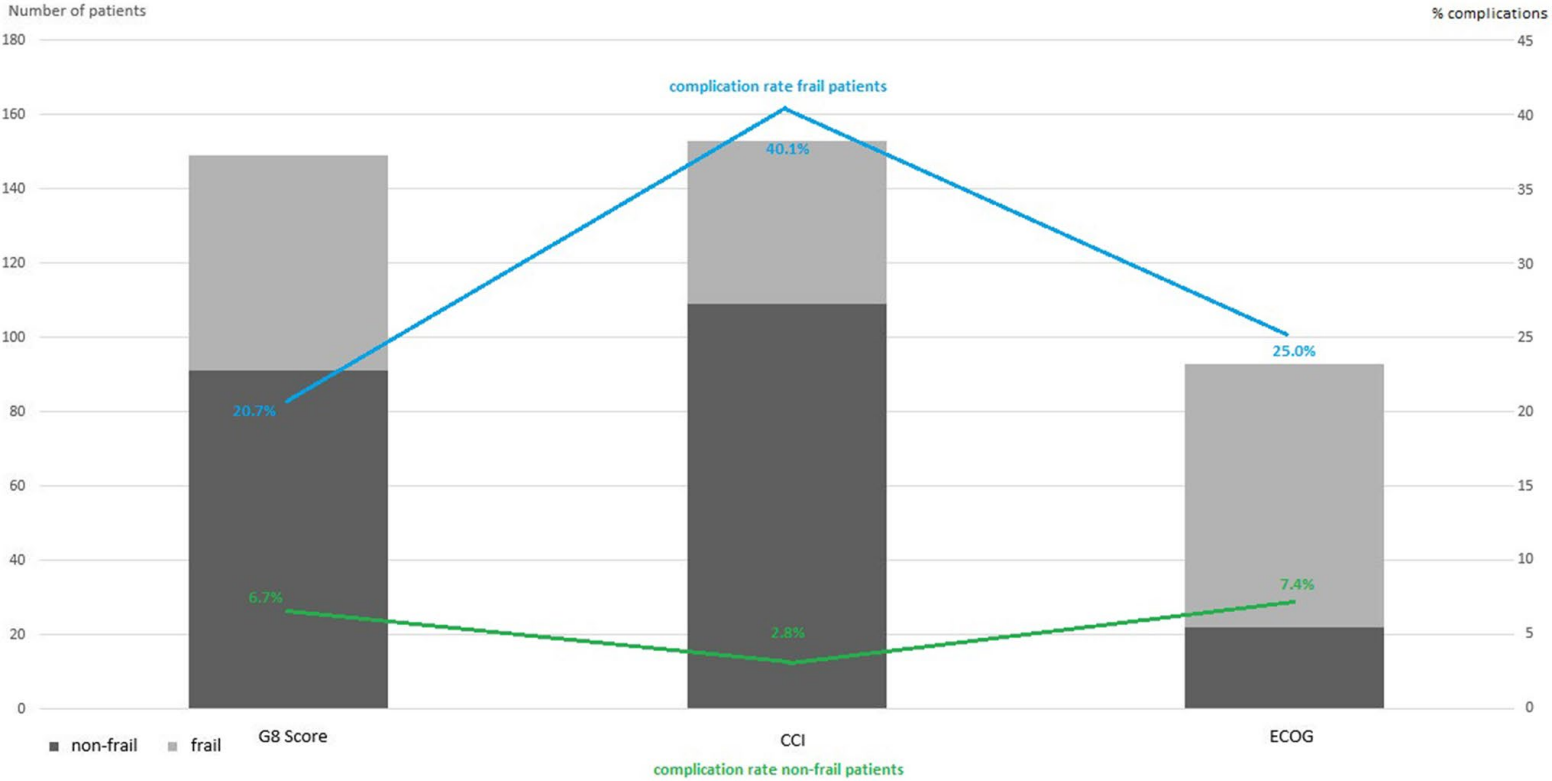
Complication rate in accordance to frailty status

Significant differences were found between the frail or non-frail group with respect to the rates of clinical postoperative complications in total (G8 Score: frail: 20.7% vs. non-frail: 6.7%, *p* = 0.028; ECOG: frail: 40.9% vs. non-frail: 2.8%, *p* = 0.002; and CCI: frail: 25.0% vs. non-frail: 7.4%, *p* = 0.003; respectively), pulmonological complications (CCI: frail: 80.0% vs. non-frail: 20.0%, *p* = 0.010), wound infections (ECOG: frail: 100.0% vs. non-frail: 0.0%, *p* < 0.001) as well as multiple complications (G8 Score: frail: 100.0% vs. non-frail: 0.0%, *p* = 0.029 and ECOG: frail:100.0% vs. non-frail 0.0%, *p* = 0.010; respectively (Fig. 2)). Additionally, the length of hospital stay was associated with preoperative ECOG and CCI frailty-status.

In propensity score matching, chronological age and BMI did not persist as a statistically significant prognostic factors for complications in the matched groups, independent of G8 Score, CCI and ECOG. The accumulation of postoperative complications did not correlate with age and BMI. Remarkably, the complication rate in the frail cohort was two to three times more common than in the non-frail cohort (G8 Score: HR: 2.12, 95%-CI: [0.012–0.313], *p* = 0.034; CCI: HR: 2.6, 95%-CI: [0.064–0.459], *p* = 0.009 and ECOG: HR: 2.6, 95%-CI: [0.087–0.617], *p* = 0.009; respectively).

## Discussion

The aim of this study was to analyze the relationship between the preoperative frailty status, measured by selected commonly used global health assessment tools, and the incidence of postoperative in-hospital complications in a highly selected patient cohort with exclusively women older than 60 years with endometrial cancer, treated in the University Hospital Mainz, Germany.

Three sub-aspects of the perioperative setting were highlighted. Related to the preoperative laboratory values, the G8 Score, ECOG and CCI showed a significant association with anemia. Surgical parameters, such as incision-suture time, drainages and intraoperative blood loss, as well as operative revisions, did not correlate with the preoperative frailty status, which proved to be a quality feature of this study, as all patients underwent the same radical surgery regardless of their preoperative global health status.

Of utmost importance, the perioperative use of blood products was highly associated with the preoperative frailty evaluation. Furthermore, postoperative clinical events, as well as the length of hospital stay correlated significantly with the preoperative frailty status raised by G8 Score and ECOG. This synergistic relationship between a higher ECOG status and the evaluation as a frail person with the G8 Score has already been demonstrated in several studies including oncological patients (Takahashi et al. 2017). One explanation for the significant correlation of the G8 Score with postoperative complications, especially in patients with EC, may be its emphasis on nutritional (three of eight items) and physical performance status (one item). The primary risk factor for developing an EC is obesity (mean BMI in our cohort is 30.19 ± 7.7), which might be perfectly reflected by the G8 Score composed of seven items of the MNA in combination with age (Emons et al. 2018; Vellas et al. 1999).

Our results are also in line with several prospective and retrospective investigations in different surgical disciplines (Birkelbach et al. 2019; Kristjansson et al. 2010; Lee et al. 2010). Birkelbach and colleagues assessed 1186 elderly patients 65 years old or older, evaluated for frailty using Fried’s 5-point frailty assessment before elective non-cardiac surgery examining its independent predictive power in the postoperative complication context (Birkelbach et al. 2019). Kristjansson et al. reported higher rates of serve complications after elective surgery for colorectal cancer in patients categorized as frail with a multidisciplinary CGA within 14 days prior to surgery (Kristjansson et al. 2010). In a series of 178 participants, they assessed the physical and cognitive functioning, comorbidity with polypharmacy, nutrition, as well as emotional status as part of a systematic CGA.

The decision to use the reported five global health assessment tools (G8 Score, ECOG, CCI, ASA PS and Lee-Index) was made considering that a larger pool of publications would enhance the study’s background and allow broader comparability. In addition, we soughed to maintain the interdisciplinarity of the frailty assessment. ECOG is a commonly used classification in oncology, whereas the ASA PS was assigned by anesthesiologists. The G8 Score and the CCI were collected in collaboration with geriatricians. Awareness of the relevance of frailty status especially in the preoperative setting and its multidimensional character could be the basis for a shared decision-making and individualized treatment. However, identifying frail cancer patients with improved risk for pre- and postoperative morbidity should include a patient-centered prehabilitation program, including nutrition counseling, physiotherapy, anticipatorily organizing postoperative home care, as well as avoiding potential preoperative complications such as hypothermia, dehydration or delirium (Mörgeli et al. 2017).

Limitations arise from the retrospective character of our study. This may be relevant especially in terms of incomplete follow-up, which were successfully reduced to a minimum of twelve patients (6.5%) by reaching out to patients and physicians through different channels of communication and an extensive review of clinical records. Moreover, the sample size, with in total 153 women was not very large, which might limit the power of the current trial. Frailty screening was offered to all women 60 years and older with EC seen in the preoperative prearrangement. We tried to minimize the possible systemic error that arises from the large range of surgical interventions and surgeons by operating all patients in one University hospital according to the current national guidelines. Outcome parameters were not rated into minor/major categories and were derived from ICD-10 coded hospital diagnoses so that limitations of routine data use were applicable. Moreover, which global health assessment tool is the most appropriate, would need to be validated in the future.

In conclusion, this is the first report to focus exclusively on elderly EC patients and the association between the preoperative frailty status measured with different global health assessment tools and perioperative parameters. In contrast to conventional prognostic parameters such as higher BMI or advanced age, the preoperative frailty status significantly correlates with preoperative anemia, perioperative transfusion rates and postoperative in-hospital complications. A standardized, possibly multidisciplinary frailty assessment in the preoperative preparation of elderly cancer patients should be established to evaluate the perioperative risk and to individualize the therapy algorithm. Further prospective research should be initiated to recognize the preoperatively frail patient in a standardized manner and to implement a patient-centered interdisciplinary discussion about individualized modified prevention and therapy management.

## Data Availability

All data generated or analysed during this study are included in this article and its supplementary material files. Further enquiries can be directed to the corresponding author.

## Abbreviations

ASA PS: American Society of Anesthesiologists Physical Status System
CCI: Charlson Comorbidity Index
CGA: Comprehensive geriatric assessment
EC: Endometrial cancer
ECOG: Eastern Cooperative Oncology Group performance status
G8 Score: G8 questionnaire
IQR: Interquartile range
MNA: Mini Nutritional Assessment
Lee-Index: Lee-Schonberg prognostic index
NSQIP: National Surgical Quality Improvement Program
SIOG: International Society of Geriatric Oncology
SD: Standard deviation
